# Reported outcomes from a community naloxone training and distribution program

**DOI:** 10.1016/j.dadr.2025.100341

**Published:** 2025-05-06

**Authors:** Kevin Frederiks, Maxwell Everett, Kristen Gilmore Powell, N. Andrew Peterson, Suzanne Borys, Donald K. Hallcom, Nina A. Cooperman

**Affiliations:** a Rutgers Robert Wood Johnson Medical School, 125 Paterson St., New Brunswick, NJ 08901, United States; b Rutgers University School of Social Work, 120 Albany St., New Brunswick, NJ 08901, United States; c Center for Prevention Science and the Northeast and Caribbean Prevention Technology Transfer Center, School of Social Work, Rutgers University, 390 George Street, 5th Floor, New Brunswick, NJ 08901, United States; d New Jersey Division of Mental Health and Addiction Services, New Jersey Department of Human Services, 5 Commerce Way, Hamilton, NJ 08691, United States

**Keywords:** Naloxone, Training, Opioid, Overdose

## Abstract

The United States opioid epidemic is an enormous public health crisis, claiming over 500,000 lives between 1999 and 2020. However, the increased availability of naloxone has saved many lives and led to the development of community-based naloxone training and distribution programs. We developed a naloxone education and distribution program in New Jersey in 2017. This program provides a 60-minute training for community members in various settings, such as police departments, community centers, etc. Participants were instructed to call the training and distribution program if they used their naloxone kit, and the program would replace it. Callers were asked a short survey about behaviors during the naloxone administration and overdose outcome. From January 2018 through June 2022, 191 calls to report an overdose and request a new kit were received. Overall, 70 (37 %) of the reported naloxone administrations were by police, 38 (20 %) family/friends, and 50 (26 %) strangers. The most common actions taken during the overdose included: 162 (85 %) calling EMS; 161 (84 %) staying with the person who overdosed until EMS arrived; and 131 (69 %) checking the individual who overdosed for signs of breathing. Individuals who helped with an overdose were able to revive the person in 172 (90 %) of the reported overdoses. Our data suggests that participants in these programs will use naloxone for opioid overdoses whether the victim is known to the participant or a stranger. Future research should focus on understanding outcomes of and behaviors during overdose episodes that are not reported to the program.

## Highlights

• One-hundred and ninety-one overdoses were reported.
• Twenty-six percent of naloxone administrations were by a stranger.
• The overdosed person was revived in 90 % of reported overdoses.
• Emergency medical services was called during 85 % of overdoses.
• Rescue breathing was provided for 31 % of overdoses.

## 1 Introduction

The United States Opioid Epidemic has represented an enormous public health crisis, claiming over 500,000 lives between 1999 and 2020 (Centers for Disease Control and Prevention, 2021). Further, opioid overdoses increased 40 % in 2020, during the COVID-19 pandemic, compared to the previous year. In 2021, the number of opioid related deaths increased six times as compared the 1999, and over 75 % of all drug overdose deaths in 2021 were opioid related (Centers for Disease Control and Prevention, 2023). In 2021, the number of opioid related deaths increased six times as compared the 1999, and over 75 % of all drug overdose deaths in 2021 were opioid related (Centers for Disease Control and Prevention, 2023). This study was conducted in New Jersey, where, from 2018 to 2022, there were 13,066 opioid-related overdose deaths, of which, 11,614 (88.9 %) were due to the synthetic opioid fentanyl (NJ Department of Health, 2024). Interventions are needed to prevent opioid overdose and save lives, especially given the potency of opioids currently available.

Naloxone is effective for reducing overdose deaths and is an agent that antagonizes the mu opioid receptor, reversing the effects of an overdose (Johansson et al., 2019). Naloxone was initially only administered intramuscularly or intravenously, and its use was limited to healthcare providers (Wermeling, 2015). However, in 2015 Naloxone was approved for use in the intranasal form, making it more widely available and expanding its use beyond the hospital setting (Lewis and Fishman, 2017). The increased availability of naloxone led to the development of community-based naloxone education and distribution programs. These programs involve educating participants on recognizing the signs of an overdose followed by instruction on naloxone use, and the distribution of naloxone to participants (Green et al., 2008). Previous research suggests that these programs are favorable and have been adopted by state agencies to help combat the opioid epidemic (Estrada et al., 2024). However, little is known about the circumstances in which naloxone kits are used and the behavior of those administering the naloxone. Further, in the new era of stronger syntenic opioids, like fentanyl, concerns have been raised about the efficacy of current naloxone dosing (Rzasa and Galinkin, 2018), and information on its dosing and the frequency with which it is effective once distributed to communities, is important to understand in the context of increased opioid potency.

Few studies have described the outcomes of a naloxone distribution program (Janssen et al., 2020). One study, describing outcomes from a naloxone training program at a County Sheriff’s department in Michigan, reported on the number of deputies trained every year (N = 508), the number of naloxone doses administered (N = 184), and the overall rate of successful naloxone administration (95 %). While this study (Janssen et al., 2020) primarily reported on the overall effectiveness of the naloxone, information about the specific behaviors taken by trained participants within the setting of an overdose, the relation of the participant to the person who overdosed, or the time between doses, when multiple doses were given, was not reported. Another study investigated whether people who inject drugs (PWID) can administer naloxone, if trained. This study found that greater than 80 % of PWID feel confident administering naloxone after receiving a short training from a physician (Piper et al., 2008).

To address the need for information on the process and effectiveness of naloxone administration in the community, we collected data on naloxone kits that were reported as used during an opioid overdose, between January 2018 and June 2022, and had been distributed by a naloxone training and distribution program, supported by the New Jersey Division of Mental Health and Addiction Services and administered by the Division of Addiction Psychiatry, Rutgers Robert Wood Johnson Medical School, Rutgers University. We assessed who administered the naloxone during the overdose and examined behaviors during and after the overdose (e.g., number of naloxone doses given, and actions taken following overdose, such as: calling emergency medical services (EMS), and providing rescue breathing, etc.).

## 2 Methods

We developed a naloxone education and distribution program that began in New Jersey in 2017. This program provides a 60-minute training program for community members in various settings, such as police departments, community centers, schools, treatment programs, and businesses, to identify the signs of an overdose and how to administer naloxone. The trainings were developed by a doctoral level clinician on our team and include information on what opioids are and where they come from, the history of the opioid epidemic, opioid overdose statistics, fentanyl, signs of an overdose versus intoxication, how naloxone works, how to administer naloxone, rescue breathings, and calling 911 (Table 1). Training content is regularly updated as the epidemic evolves (e.g., information on fentanyl and xylazine). The trainings are administered by staff with bachelor’s or master’s degrees in social work or public health. Participants are provided with a free naloxone kit that includes two doses of 4 mg intranasal naloxone, a rescue breathing shield, nitrile gloves, and a storage pouch. The kit has the programs’ name and phone number printed on the pouch with instructions to call for a replacement kit when the kit is used or expired. Each kit is numbered so that the program knows at what training the kit was distributed. When a person calls to report a used kit, program staff ask about the relationship between the person who administered the naloxone and the person who overdosed, total number of naloxone doses administered, whether the naloxone was effective in reversing overdose, length of time between naloxone doses, if multiple doses were given, and behaviors taken by individuals that witnessed the overdose. Data reported in this study were collected from January 2018 through June 2022. Survey data were entered into Microsoft Excel (Version 16.67) and cross-checked. Data were then analyzed in Excel and SPSS 29. The study was reviewed by the Rutgers Institutional Review Board.

**Table 1:** Training content.

Definition of opioid drugs and their effects
Opioid use and overdose epidemic statistics
History of the opioid crisis and how it happened
Prescription opioids and illicit opioids
Fentanyl
Signs of an opioid use disorder
Risk factors for opioid overdose
Signs of opioid intoxication versus opioid overdose
Instructions to call 911
Information on the Opioid Antidote and Overdose Prevention Act
What naloxone is and how it works to reverse overdose
Instructions for administering naloxone
Steps for rescue breathing
Opioid withdrawal symptoms
Withdrawal and re-overdose risk post naloxone administration
Resources for treatment
Additional ways to access naloxone
Instructions for storing naloxone
Do’s and don’ts when responding to an opioid overdose
Instructions on how to replace a used or expired naloxone kit

## 3 Results

In total, 15,953 people attended 1667 naloxone trainings and 16,429 naloxone kits were distributed from January, 2018--June, 2022. Overdoses and used naloxone kits were reported 191 times (Table 2). Naloxone was administered by police (37 %, n = 70), strangers (26 %, n = 50), family members (14 %, n = 27), friends (6 %, n = 11), or a staff member in a social service or clinical setting (12 %, n = 23), or an unknown individual (5 %, n = 10). Deployed kits were distributed at police departments (n = 68; 35 %), monthly regional trainings open to the public (n = 28; 15 %), clinical settings (n = 15; 8 %), schools and universities (n = 15, 8.4 %), or other settings (e.g., health departments or other community organizations; n = 65; 34 %). Actions taken during an overdose included 85 % (n = 162) calling EMS, 84 % (n = 161) staying with the person who overdosed until EMS arrived, 69 % (n = 131) checking the individual who overdosed for signs of breathing, 12 % (n = 23) checking the person’s airway, and,19 % (n = 37) putting the person who overdosed in the recovery position. Most who administered naloxone provided 2 or more doses (n = 120; 64 %). Time between the first and second doses, among those who administered more than one dose, varied, with 17 % (n = 20) < 3 minutes, 28 % (n = 34) between 3 and 5 minutes, and 23 % (n = 28) > 5. The person who had overdosed was revived in 90 % (n = 171) of the cases. Six percent of those who overdosed was known to have died. The person not being revived, but the outcome was unknown for 4 % of the overdoses (n = 8, e.g. EMS brought the person to the hospital before the outcome was known).

**Table 2:** Characteristics of naloxone Use (N = 191).

**Person Who Administered the Naloxone**	**n (%)**
Police	70 (37)
Stranger	50 (26)
Family Member	27 (14)
Friend	11 (6)
Clinical/Social Service Program Staff	23 (12)
Unknown	10 (5)
**Setting Where Deployed Naloxone Kit Distributed**	
Regional trainings open to the public	28 (15)
Police departments	68 (36)
Clinical settings	15 (8)
Schools and universities	15 (8)
Other^a^	65 (34)
**Number of Naloxone Doses Administered**^b^	
1	68 (36)
2	108 (57)
3	9 (5)
4	3 (2)
Unknown	3 (2)
**Time Between Naloxone Doses**^c^	
<1 Minute	5 (4)
1–3 Minutes	15 (13)
3–5 Minutes	34 (28)
> 5 Minutes	28 (23)
Unknown	38 (32)
**Actions Taken During the Overdose**^d^	
EMS Called	162 (85)
Stayed Until EMS Arrived	161 (84)
Provided Rescue Breathing	59 (31)
Put in Recovery Position Checked for signs of breathing Checked airway	37 (19) 131 (69) 23 (12)
**Outcome of Naloxone Administration**	
Person Passed Away	11 (6)
Person Known Revived	172 (90)
Not Revived, Not Known Dead	8 (4)

a Including health departments and other social service or community organizations.

b Doses other than those given to a particular individual during the training may have been used.

c Among those who administered more than one dose.

d Instructions provided during the training.

## 4 Discussion

This study showed that naloxone training and distribution programs can educate communities and provide needed resources. Individuals were trained in various community settings, including schools, health departments, police departments, libraries, churches, treatment programs, and shelters. Naloxone was administered by police, strangers, friends, and family. Most called and stayed with the person who overdosed until EMS arrived and checked the individual who overdosed for signs of breathing. Even as opioids were becoming more potent with fentanyl, we know that 172 lives were saved by naloxone distributed from our program.

Further, our data suggests that participants in naloxone training programs will use naloxone for opioid overdoses whether the person who overdosed is known to the participant or a stranger. This reinforces the potential of these programs, as previous research has suggested that individuals are most often reviving a friend or relative rather than a stranger (Katzman et al., 2020, Doe-Simkins et al., 2014).

Almost all who overdosed (90 %) were revived. Our findings are consistent with previous studies that have estimated a 75–100 % effectiveness of naloxone in reversing overdose (Estrada et al., 2024, Fischer et al., 2025, Green et al., 2008). This result of 90 % success rate is reassuring in the current opioid environment, with stronger formulations such as fentanyl having become more available, and concerns having been raised about the efficacy of current naloxone dosing for opioid overdose reversal (Rzasa and Galinkin, 2018). Our results are consistent with other studies that suggest that current naloxone dosing is still as effective, even in the fentanyl era (Carpenter et al., 2020, Rock et al., 2024). However, under circumstances when the person overdosing was not revived, those using the naloxone may have been less motivated to call the program and request an additional kit. More research is necessary to identify factors associated with naloxone not working.

The community-based naloxone training supported behaviors, besides administering naloxone, that would be helpful for participants to perform within the setting of an opioid overdose (e.g., calling EMS, staying with the person who overdosed and checking for signs of breathing). The majority who administered naloxone called EMS and stayed with the person who overdosed until help arrived. However, in a minority of cases, rescue breathing, checking the overdosed individual’s airway, or putting the individual in the recovery position was performed. These results are consistent with another study by Doe-Simkins et al. (2014) that reported behaviors during naloxone administration like staying with the victim until help arrived (89 %) and performing rescue breathing (47 %). Although rescue breathing and putting the person in the recovery position were covered in the training, possibly, these behaviors were less likely to occur because they require contact with the overdosed individual. Further research is needed to identify and address barriers to these behaviors.

We also found that 67 % of participants administered more than one dose of naloxone, compared to 48 % of trained participants giving 2 or more doses reported by Doe-Simkins et al. (2014). This could be due to the rise of more potent opioids (e.g., fentanyl) in the drug supply during the period of our study (Palamar et al., 2024). Additionally, we found that, of individuals administering > 1 dose, 51 % waited > 3 minutes to administer the second dose, despite current guidelines recommending second doses of naloxone if there is no response within 2–3 minutes (Regina et al., 2025). Although participants were instructed to wait 2–3 minutes during the training, a large percentage of individuals may have waited longer given that some fact sheets recommended waiting 2–5 minutes between doses during the study time-period (NJ Division of Mental Health and Addiction Services, 2017).

While our study suggests a benefit of community-based naloxone programs for preventing opioid overdoses in the community, there are limitations to this study. Data were only collected from overdoses where individuals reached out to us to request a replacement for their naloxone kit. Therefore, we did not capture data from the overdoses that were not reported to us. Also, our study was observational, and we are unable to determine the benefits of the naloxone training versus naloxone distribution (as both occurred during the same session). Finally, these data were based on self-report by someone who called our program for a replacement kit; the reporter may have been the person who administered the naloxone, an observer of the person who administered the naloxone, or a reporter of information that they received second hand.

Despite these limitations, our data provides preliminary information on the role community-based naloxone distribution programs can play in preventing overdose deaths and indicates that lives can be saved through naloxone distribution and overdose prevention training. Future research should focus on understanding whether the training of community members in combination with naloxone distribution decreases overdoses, or if increased access to naloxone alone decreases overdoses.

## CRediT authorship contribution statement

**Frederiks Kevin:** Writing – review & editing, Writing – original draft, Formal analysis, Conceptualization. **Powell Kristen Gilmore:** Writing – review & editing. **Everett Maxwell:** Writing – original draft, Data curation, Conceptualization. **Borys Suzanne:** Writing – review & editing, Project administration, Funding acquisition, Conceptualization. **Peterson N. Andrew:** Writing – review & editing. **Cooperman Nina:** Writing – review & editing, Writing – original draft, Project administration, Data curation, Conceptualization. **Hallcom Donald K.:** Project administration, Conceptualization.

## Author disclosure

Nina Cooperman reports financial support was provided by New Jersey Division of Mental Health and Addiction Services. If there are other authors, they declare that they have no known competing financial interests or personal relationships that could have appeared to influence the work reported in this paper.

## Declaration of Competing Interest

The authors declare the following financial interests/personal relationships which may be considered as potential competing interests: Nina Cooperman reports financial support was provided by New Jersey Division of Mental Health and Addiction Services. If there are other authors, they declare that they have no known competing financial interests or personal relationships that could have appeared to influence the work reported in this paper.
